# A new bioinspired method for pressure and flow sensing based on the underwater air-retaining surface of the backswimmer *Notonecta*

**DOI:** 10.3762/bjnano.9.282

**Published:** 2018-12-14

**Authors:** Matthias Mail, Adrian Klein, Horst Bleckmann, Anke Schmitz, Torsten Scherer, Peter T Rühr, Goran Lovric, Robin Fröhlingsdorf, Stanislav N Gorb, Wilhelm Barthlott

**Affiliations:** 1 Nees Institute for Biodiversity of Plants, University of Bonn, Venusbergweg 22, D-53115 Bonn, Germany; 10 Swiss Light Source, Paul Scherrer Institute, 5232 Villigen, Switzerland; 11 Department of Functional Morphology and Biomechanics, Institute of Zoology, Christian-Albrechts-Universität zu Kiel, Am Botanischen Garten 1–9, D-24118 Kiel, Germany; 2 Institute of Crop Science and Resource Conservation (INRES) – Horticultural Science, University of Bonn, Auf dem Hügel 6, D-53121 Bonn, Germany; 3 Institute of Nanotechnology (INT), Karlsruhe Institute of Technology (KIT), Hermann-von-Helmholtz-Platz 1, D-76344 Eggenstein-Leopoldshafen, Germany; 4 Institute of Applied Physics (APH), Karlsruhe Institute of Technology (KIT), Wolfgang-Gaede-Strasse 1, D-76131 Karlsruhe, Germany; 5 Institute for Zoology, University of Bonn, Meckenheimer Allee 169, D-53115 Bonn, Germany; 6 Karlsruhe Nano Micro Facility (KNMF), Karlsruhe Institute of Technology (KIT), Hermann-von-Helmholtz-Platz 1, D-76344 Eggenstein-Leopoldshafen, Germany; 7 Institute of Zoology, Biocenter, University of Cologne, Zülpicher Straße 47b, D-50674 Cologne, Germany; 8 Centre for Molecular Biodiversity Research (zmb), Zoological Research Museum Alexander Koenig (ZFMK), Adenauerallee 160, D-53113 Bonn, Germany; 9 Centre d’Imagerie BioMédicale, École Polytechnique Fédérale de Lausanne, 1015 Lausanne, Switzerland

**Keywords:** mechanoreceptor, *Notonecta* sensor, pressure sensor, Salvinia effect, superhydrophobic surfaces

## Abstract

In technical systems, static pressure and pressure changes are usually measured with piezoelectric materials or solid membranes. In this paper, we suggest a new biomimetic principle based on thin air layers that can be used to measure underwater pressure changes. Submerged backswimmers (*Notonecta sp.*) are well known for their ability to retain air layers on the surface of their forewings (hemelytra). While analyzing the hemelytra of *Notonecta*, we found that the air layer on the hemelytra, in combination with various types of mechanosensitive hairs (clubs and pins), most likely serve a sensory function. We suggest that this predatory aquatic insect can detect pressure changes and water movements by sensing volume changes of the air layer under water. In the present study, we used a variety of microscopy techniques to investigate the fine structure of the hemelytra. Furthermore, we provide a biomimetic proof of principle to validate our hypothesis. The suggested sensory principle has never been documented before and is not only of interest for sensory biologists but can also be used for the development of highly sensitive underwater acoustic or seismographic sensory systems.

## Introduction

**Figure 1:**
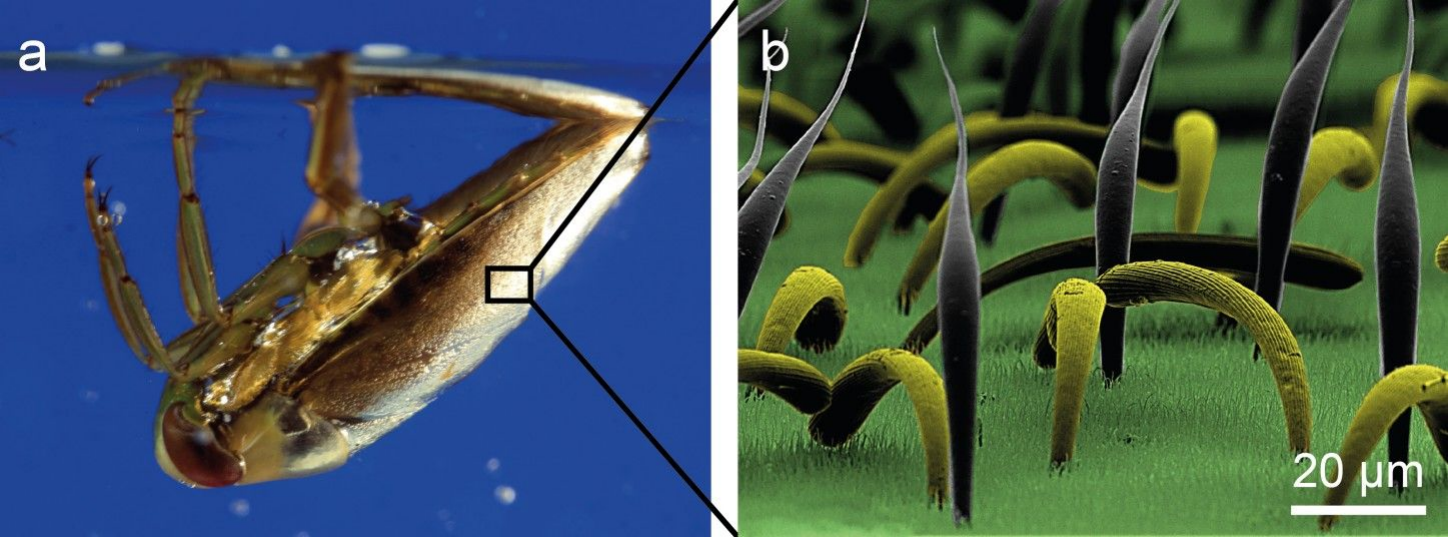
a) The backswimmer *N. glauca*. The silvery shine on the surface of the hemelytra is caused by the total reflection of light at the air–water interface. b) Colored SEM-image of the surface of the hemelytra of a backswimmer. Two types of setae (“clubs” are yellow and “pins” are grey) and a “carpet” of short, densely packed “microtrichia” stabilize the air layer and assure stable air retention. Source: (b) modified after [11].

The surfaces of animals and plants are interfaces between the organisms and the environment. Since animals and plants inhabit many different environments, it is not surprising that over the course of about 3.7 billion years of biological evolution [1–3], a stunning diversity of surface architectures has evolved. Today, millions of living prototypes (species) exist, waiting to be used for the development of biomimetic technical innovations [4–5]. Well know examples are insect adhesive pads [6] or the structural colors of *Morpho menelaus* [7]. Superhydrophobic surfaces are also important in the above context. Several plants and animals, which can maintain stable air layers while submerged (Salvinia effect [8]), have been analyzed. Especially the floating ferns of the genus *Salvinia* (Salviniales: Salviniacae) and the backswimmer *Notonecta* (Hemiptera: Notonectidae) (Figure 1a) have been shown to be ideal model organisms for the development of biomimetic air-retaining surfaces [4,9–10]. Thus, stable air layers bear a high potential for biomimetic technical applications, e.g., for drag reducing ship coatings [5].

Submerged backswimmers are covered with a thin air layer, in particular on their hemelytra (forewings) [4,11–12]. This air layer remains stable over long periods of time under both static and dynamic conditions [4,13–15]. To understand the mechanism that *Notonecta* use to maintain this air layer while submerged, Ditsche-Kuru et al. [12] investigated the micro- and nanostructure of the hemelytra of *Notonecta glauca*. They found that the upper side of the hemelytra is hierarchically structured by two types of setae and many microtrichia. One type of setae is tapered and bent and the tip points in an anterior-distal direction (Figure 1b). The other type is clubbed, the tip of this type points in a posterior direction (Figure 1b) [12].

In the present study, we investigated the micromorphology and innervation pattern of the two types of setae of *Notonecta glauca* and *Notonecta maculata*. In addition, we studied the mechanical interaction between the air-covered hemelytra, the setae and the surrounding water, especially in the case of pressure changes. This part of our study suggests that *Notonecta* can use its setae to detect pressure changes, e.g., those caused by a prey animal passing by. Thorpe and Crisp [16] suggested that the benthic water bug, *Aphelocheirus aestivalis,* uses the setae on its surface – in combination with thin air layers – to measure static pressure, but the use of air layers for the detection of rapid pressure changes, i.e., for prey detection, has never been suggested before. To learn whether *Notonecta* can detect a fish passing by without visual and surface wave cues, behavioral experiments were performed. Finally, we provide a biomimetic proof of concept of the suggested principle.

## Results and Discussion

**Figure 2:**
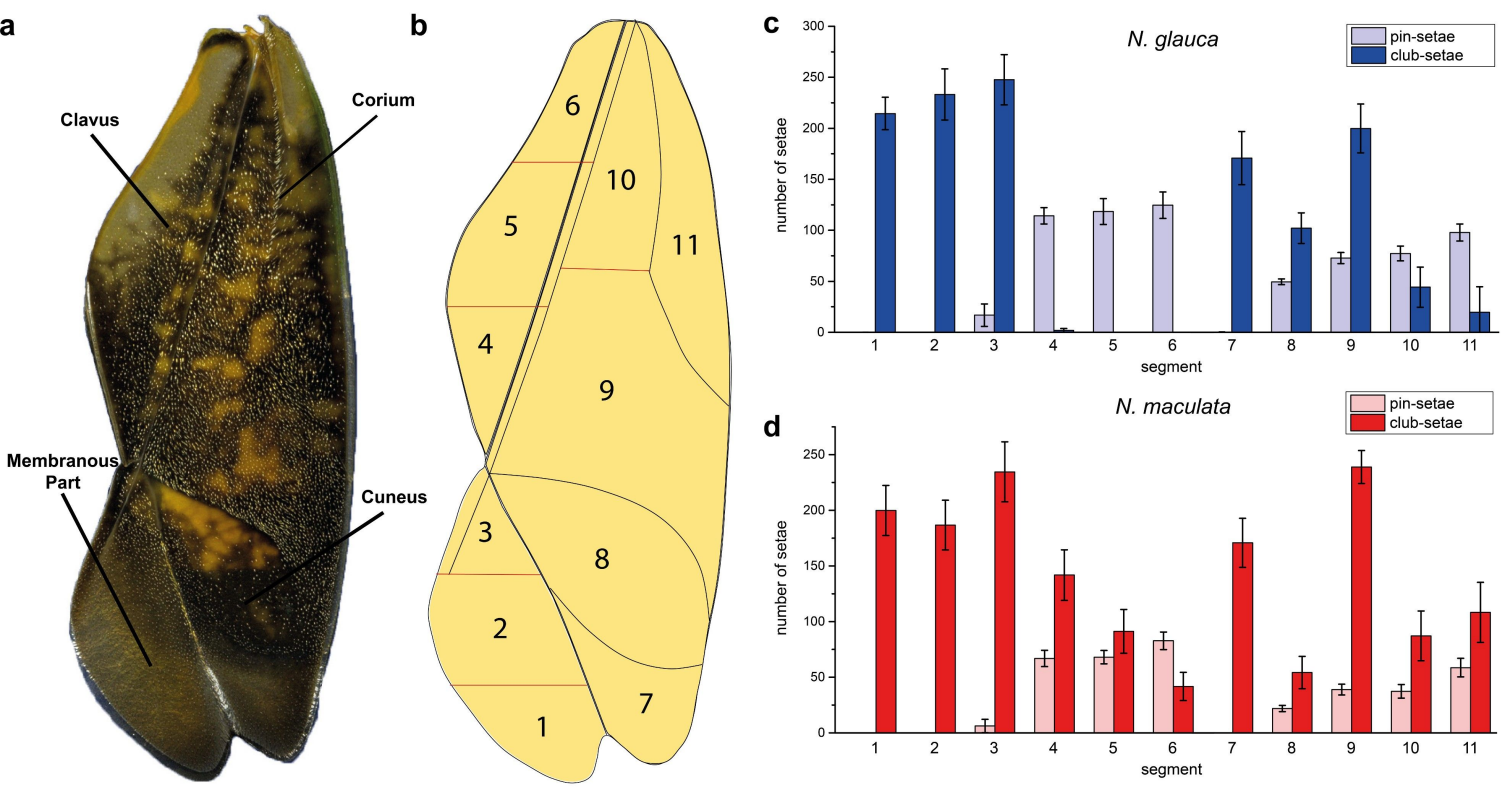
a) Left hemelytron of the backswimmer *N. glauca*. Four sections, defined by Wachmann [17], are shown. b) Scheme of the left hemelytron subdivided in 11 sections, according to Wachmann [17], and to variations in the hair density. c) Number of setae on the hemelytra of *N. glauca* and distribution of pins and clubs within the 11 sections shown in (b). d) Number of setae on the hemelytra of *N. maculata* and distribution of pins and clubs within the 11 sections shown in (b).

The hemelytra of *Notonecta glauca* (Linnaeus 1758) and *N. maculata* (Fabricius 1794) were investigated. As already described [12], the hemelytra of *Notonecta* are covered with many setae. Counting the number of setae in several arbitrarily chosen surface areas, we could extrapolate some 6,500 setae in total on each hemelytron. Both the number and the distribution of setae showed differences across 11 surface sections, defined according to Wachmann [17], and to variations in the hair density (Figure 2). Both *Notonecta* species differed slightly in the number and distribution of setae.

**Figure 3:**
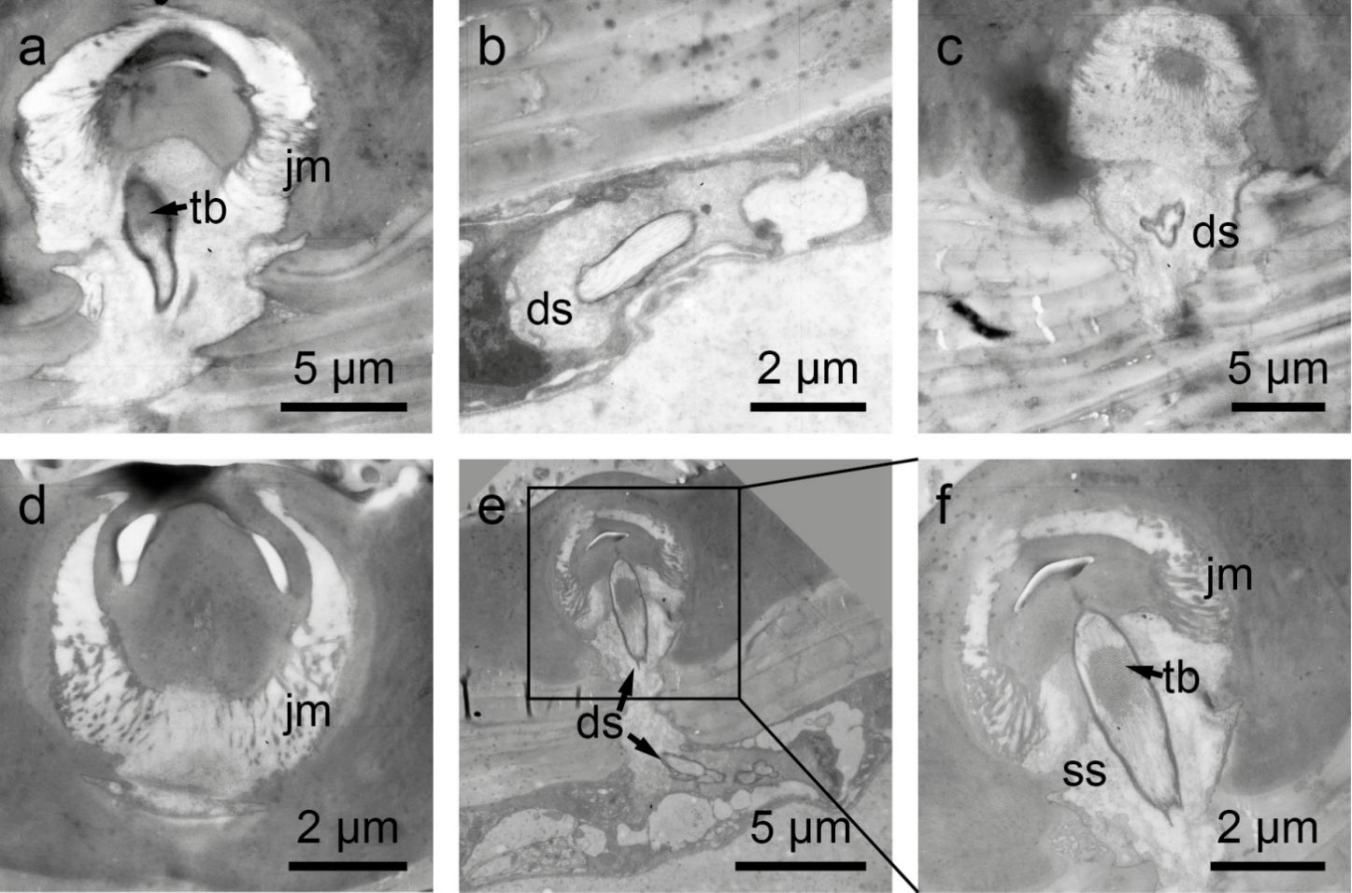
Transmission electron microscopy images of setae on the hemelytra of *N. glauca*. a–d) Clavus. a) Tubular body (tb) at the base of the seta. Note the joint membrane (jm). b) Outer dendritic segment with the dendritic sheath (ds) sectioned below the cuticle. c) Part of the dendrite in the outer receptor lymph cavity of the sensillum. The base of the seta is visible above the dendrite. d) Base of the seta with the joint membrane (jm). e,f) Membranous part of the hemelytra. e) Dendrite with an apical tubular body attached to the base of the seta. Below more proximal parts of the dendrite are shown. f) Detail of e. The tubular body (tb) is visible. The socket septum (ss) is attached to the tip of the dendrite where the tubular body is located.

Transmission electron microscopy (TEM) revealed that each seta of the *Notonecta* hemelytra was part of a sensory complex consisting of (1) a tubular body, (2) a joint membrane and (3) an outer dendritic segment with a dendritic sheath (Figure 3). All sectioned sensillae (*n* = 15) were hair mechanoreceptors with a well-developed tubular body, a joint membrane and a socket septum (Figure 3). The dendrite is enveloped in a dendritic sheath whose base shows a ciliary constriction and runs into the soma region of the sensillum (Figure 3). Tomography images using focused ion beam (FIB) techniques revealed that the two types of setae differ in micromorphology (Supporting Information File 1), most likely indicating different functions.

**Figure 4:**
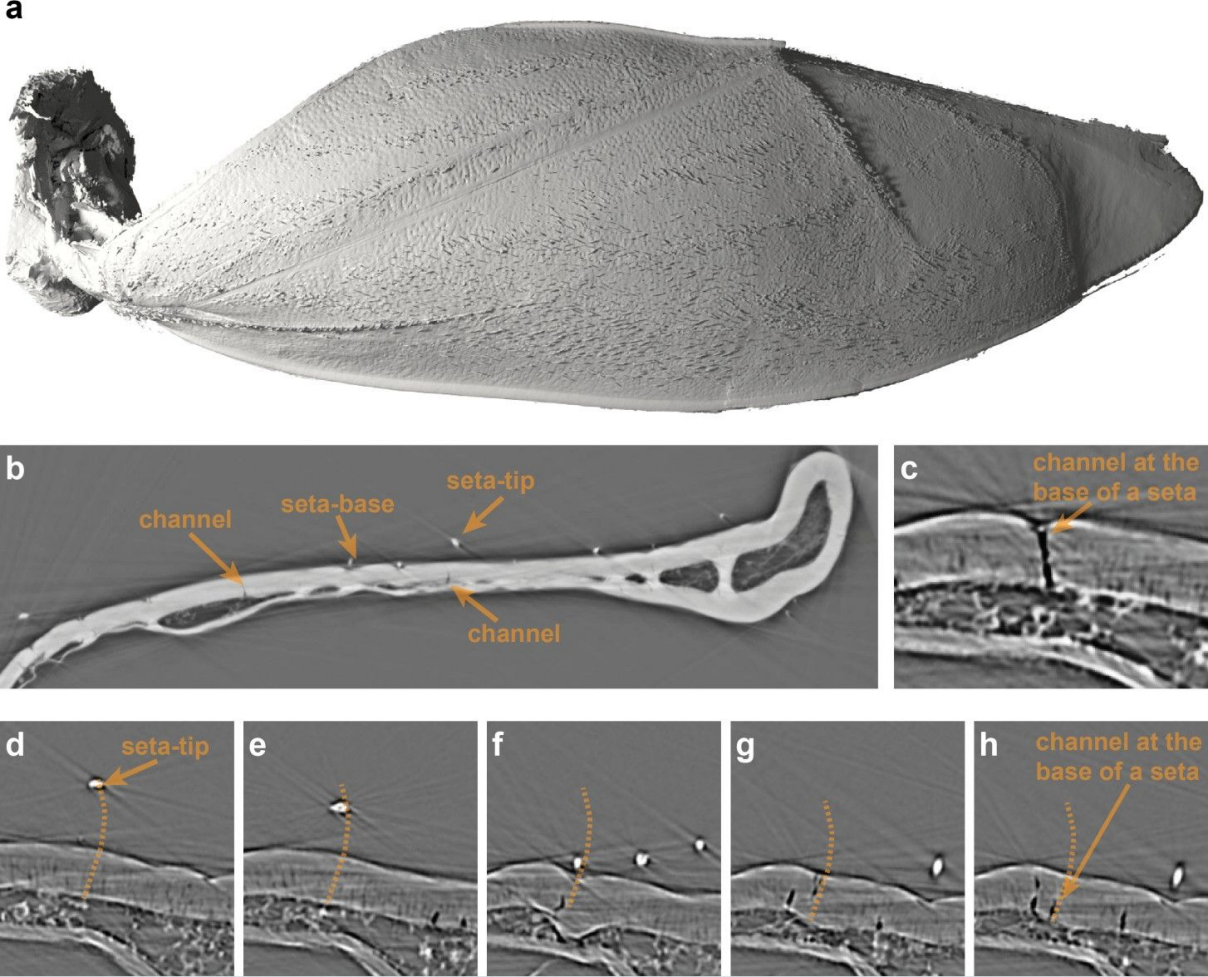
a) Three-dimensional reconstruction of a hemelytron µCT scan. The tomography data allowed an analysis of the outer and inner structures of the hemelytron. b) Digital cross section through a SR-µCT scan of a hemelytron. c) Part of the cross section, where the dendritic canal below the setal base is visible, indicating an innervation of the seta. d–h) A stack of µCT-images used for tracking single seta and identifying the corresponding setal base and setal tip.

**Figure 5:**
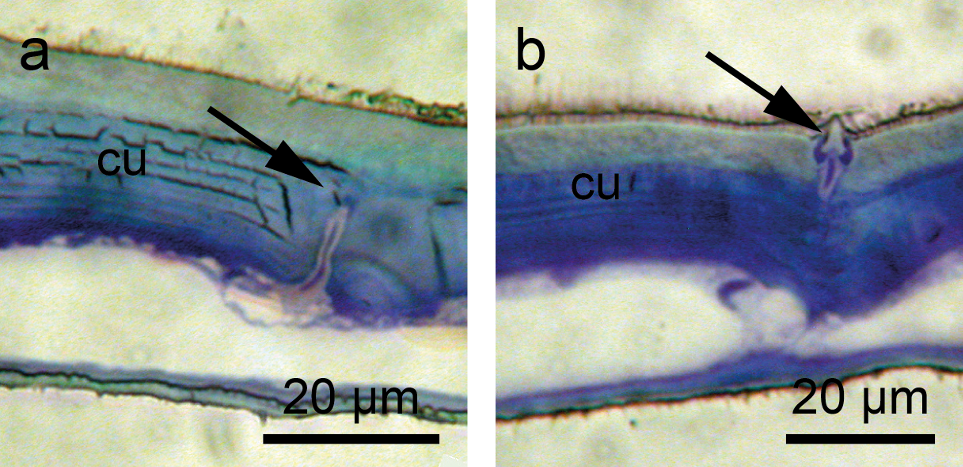
Toluidine blue/borax stained semi-thin sections through the clavus of a hemelytron of *N. glauca* (dorsal surface is up). Arrows mark mechanoreceptors. a) Lower part of a dendritic canal running through the cuticle with an inner dendrite. b) Socket region of the seta with outer dendritic tip at its base. cu = cuticle.

To determine whether the setae are innervated by nerve cells, various areas on an entire hemelytron were analyzed by microtomography (µCT) and synchrotron-microtomography (SR-µCT). The three-dimensional reconstruction of an entire hemelytron analyzed by µCT allowed the identification of every single seta and its dendritic canal on the hemelytron (Figure 4). The data suggest that probably all 6,500 setae on one hemelytron are contacted by nerve cells. In light microscopic images of stained hemelytra samples, the typical morphological features of mechanoreceptors, such as a dendritic canal or an outer dendritic tip, were identified (Figure 5).

**Figure 6:**
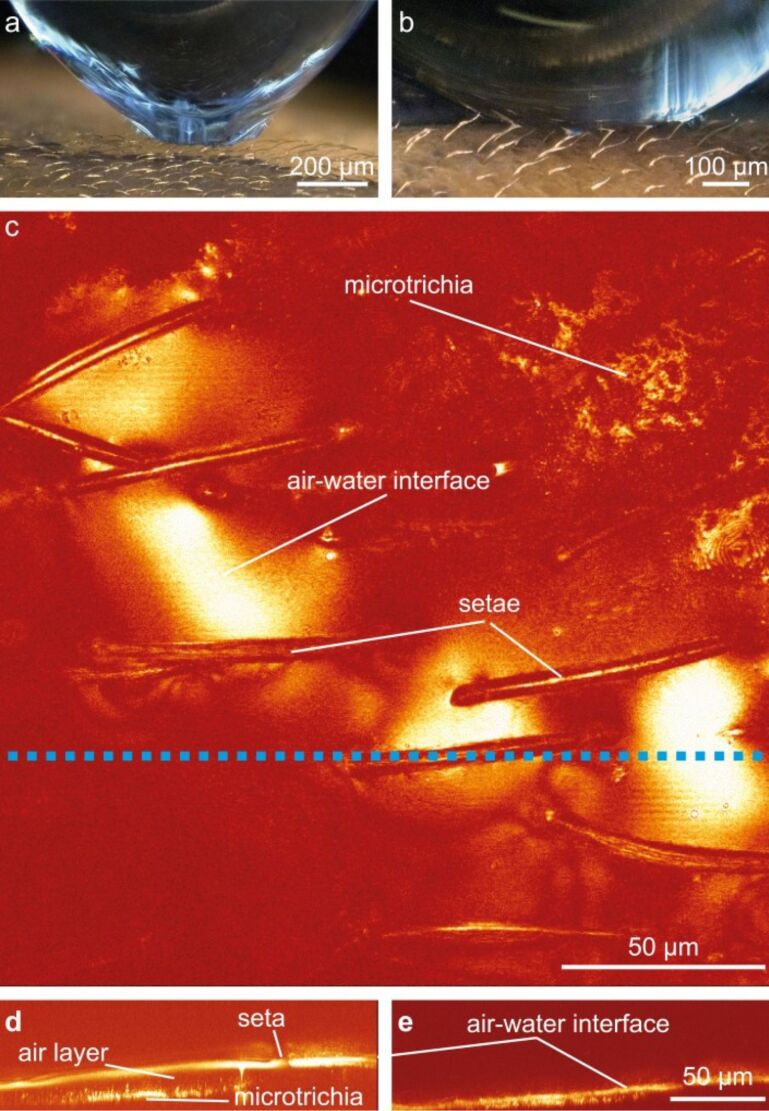
a) Image of a water droplet detaching from the surface of *N. glauca*. The deformation of the droplet indicates adhesion between the droplet and the pin-setae. This suggests that the pin-setae penetrate the air–water interface and become wet. b) Water droplet lying on the club-setae of the superhydrophobic surface of *N. glauca*. c) Projection through a stack of images using confocal laser scanning microscopy. The picture shows the structure of the setae and the reflecting air layer in between the club-setae. d) Cross section through the image stack at the position of the blue line in (c) at ambient pressure. The shape of the air–water interface and the position of the club-setae and the microtrichia are visible. e) Cross section at the same position at a pressure of 180 mbar. The air layer is compressed and the air–water interface is lying on the microtrichia. The clubs are deflected towards the surface.

The morphological data suggest that the clubs (Figure 1b, yellow) are used for pressure detection while the pins (Figure 1b, grey) are used for the detection of drag caused by water flow. To verify this hypothesis, a droplet of water was put on the surface of a hemelytron. Images of this droplet were taken using a digital video microscope (Keyence VHX 1000). Figure 6a shows a droplet that becomes detached from the surface. The strong deformation of the droplet indicates high adhesive forces, in turn indicating that the pins became stuck inside the droplet. When the droplet lies on the surface, the clubs support the droplet and prevent surface wetting of the hemelytron (Figure 6b).

**Figure 7:**
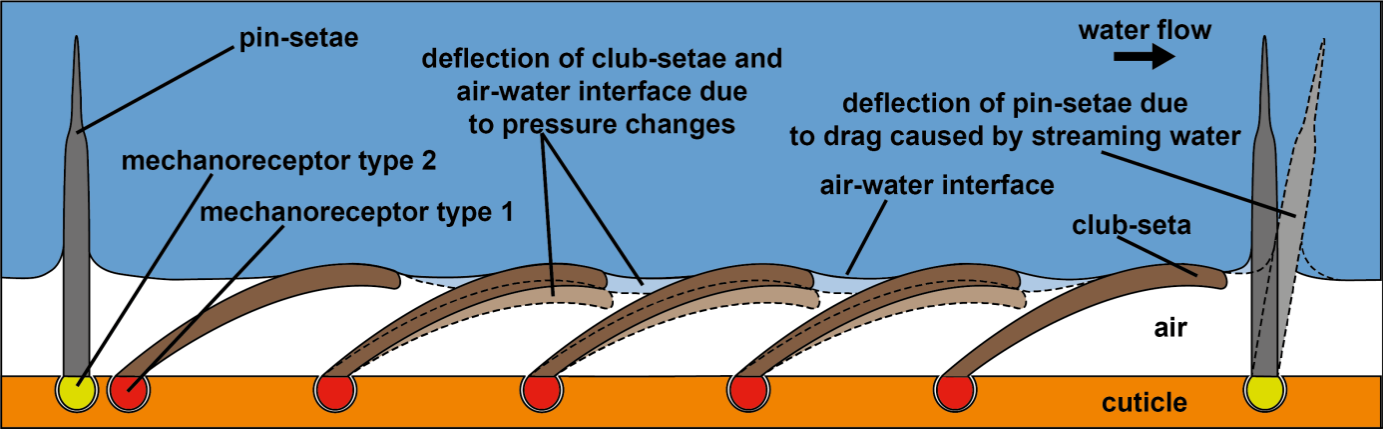
Proposed *Notonecta* forewing surface function. An air layer is kept in between the setae. The club-setae (dark brown) support the air–water interface. If pressure increases, the air is compressed and the air–water interface is deformed. This deflects the seta (light brown, dashed outline). Mechanoreceptors (red) connected with the cuticle (orange) at the base of each seta should enable monitoring of the setal deflection. The pins (dark gray) most likely penetrate the air–water interface. If so they should be deflected (light gray, dashed outline) by water flow. This deflection most likely is sensed by cuticular mechanoreceptors (yellow).

The results so far suggest that *Notonecta* uses air layers in combination with mechanosensitive setae not only for drag reduction, but also for the detection of prey or predators. With one exception [16], the involvement of air layers in a sensory function has never been demonstrated. A possible principle for a sensor that uses an air layer for the detection of pressure changes is shown in Figure 7. Since water can be considered as incompressible, a pressure wave that impinges on an air layer compresses the air. If so, the air–water interface is deformed and the club-setae, i.e., the setae that hold the air layer, are deflected due to surface tension. The mechanosensitive cells at the base of each seta most likely detect this deflection. This should enable backswimmers to sense minute pressure changes. If a spherical pressure wave propagates through the water, different setae should be deflected with certain time delays, depending on the direction and velocity of the spherical pressure wave. The second type of setae, the pins, penetrate the air–water interface and thus should be in direct contact with the water outside the air layer. We suggest that any water flow in the vicinity of a backswimmer deflects the pins and thus is detected by *Notonecta*.

To find out whether a deflection of the setae indeed occurs if pressure changes, confocal laser scanning microscopy (CLSM) was used (see Experimental section). In the projection through a stack of CLSM images, the setae as well as the reflecting air–water interface in between the setae could be monitored (Figure 6c). Cross sections through image stacks taken at different pressure amplitudes show a deflection of the setae (Figure 6d,e). This again suggests that the setae of *Notonecta* may have a sensory function.

The hypothesis that *Notonecta* uses an air layer as part of a sensory system that detects pressure changes and water movements is further supported by preliminary behavioral experiments. In these experiments, submerged *Notonecta* (N = 25) caught small fish passing by (Supporting Information File 2). As experiments were performed in complete darkness, visual cues were excluded. Furthermore, cork pieces covering the water surface excluded surface wave detection. We cannot, however, rule out that *Notonecta* has used its leg mechanoreceptors to detect the passing fish. Due to the large distance between the leg tips and the passing fish we believe that this is highly unlikely. Backswimmers only tried to catch a fish if it moved its tail fin and if the distance to the fish was ≤1.8 body length. In these cases, the backswimmer attempted to catch the fish in about 90% of the cases.

**Figure 8:**
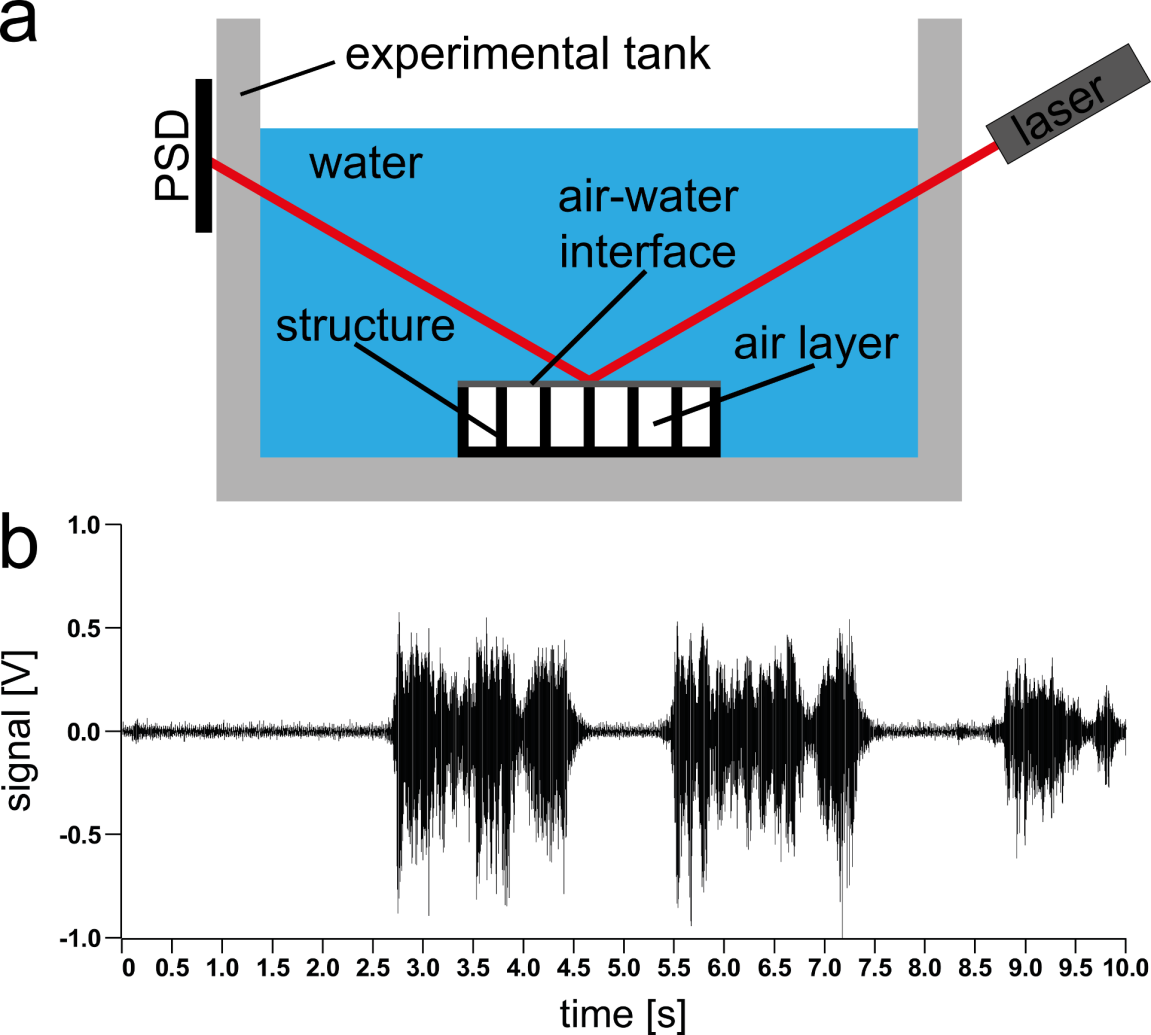
a) Setup used for the proof of concept for the biomimetic *Notonecta* sensor inspired by the backswimmer hemelytra surface. An air-retaining surface was placed at the bottom of a water-filled aquarium. A laser beam hit the air–water interface at an angle of about 30°. The reflected beam was detected by a photosensitive diode (PSD) placed at the opposite side of the aquarium. A temporary displacement of the air–water interface due to pressure changes led to beam deflection, thus the diode signal could be used to detect vibrations at the interface. b) Diode signal recorded in the experiment described in (a). The graph shows the output voltage of the diode which corresponds to the operators talking in front of the aquarium.

Additional support for a possible sensory function of the air layer on the backswimmer hemelytra surface was provided by an experiment combining a technical air-retaining surface and an optical sensor (for details see Experimental section). With this setup (Figure 8), which represents a biomimetic proof of principle, we were able to record the verbal conversation of two persons standing in front of the experimental tank. This was the final proof that under water pressure changes can be detected with aid of thin air layers.

## Conclusion

By investigating the setae on the forewings (hemelytra) of backswimmers, we found that *Notonecta* may use a thin air layer – kept under water by a hierarchically structured surface – for the detection of prey. Micromorphological investigations of the pin- and club-setae revealed morphological attributes of mechanoreceptors suggesting that backswimmers can sense the deflection of these setae. This deflection is achieved by a compression of the air layer, a hypothesis that is in line with our preliminary behavioral experiments.

Although highly complex and elaborated sensory systems are well known in biology, a sensory system based on a submerged air-retaining surface has – with a single exception [16] – never been suggested before. The functionality of the suggested *Notonecta* sensor principle has been shown by a biomimetic proof of principle. Electrophysiological and further behavioral experiments are needed for the final proof that *Notonecta* indeed employs the suggested sensory principle for prey detection. In any case, the suggested sensory system has a strong potential for the development of highly precise underwater sensors.

## Experimental

### Animals

Adult *Notonecta glauca* and *N. maculata*, caught between 2011 and 2017 in a semi-natural pond (“Melbweiher”) in the Botanical Garden of the University of Bonn, have been studied. For our experiments, the hemelytra of *Notonecta* were either fresh, fixed for electron microscopy, air dried or critical point dried (Balzers CPD 020, Bal Tec AG).

### Statistical analysis of setae distribution

A digital video microscope (Keyence VHX 1000) was used to analyze three left and two right hemelytra of *N. glauca* and *N. maculata*. The hemelytra were subdivided into 11 segments (Figure 2b) and in each segment the number of setae was counted in three randomly selected areas of 0.5 mm^2^.

### CLSM investigations of the pressure behavior

The air–water interface was analyzed by confocal laser scanning microscopy (CLSM, Leica TSM 500). The laser beam of the microscope was reflected at the air–water interface allowing a three dimensional reconstruction of that interface (see [18]). The samples were placed in a custom pressure cell allowing defined pressure changes while imaging the air–water interface (Figure 6).

### Microstructure of the setal bases

Different methods were used to analyze the microstructure of the setal bases.

The hemelytra were cut into several pieces. The pieces were fixed in 2.5% glutaraldehyde and 1.5% osmium tetroxide in cacodylate buffer (380 mOsmol, pH 7.1). After dehydrating in ethanol, the pieces were embedded in Epon 812 via epoxy propane as an intermedium. The clavus and the membranous part of the hemelytra, both situated at the inner part of the hemelytra, were sectioned. Semi-thin and ultrathin sections were prepared using a histo-diamond-knife (Diatome). Ultrathin sections were contrasted with lead citrate and uranyl acetate and evaluated with a Zeiss EM 109. Semi-thin sections were stained with 0.1% toluidin blue/borax solution and photographed using a Leitz Dialux 20 and a Nikon coolpix 5000. The images shown in Figure 3 suggest that the setal bases are mechanosensitive. Figure 5 also provides evidence for mechanoreceptors and allows a more detailed view on the microstructures.

Furthermore, the microstructures of the setal bases were analyzed using focused ion beam techniques (FIB, Zeiss Auriga 60). In this case, fresh hemelytra were covered with a thin gold layer using a sputter coater (Sputter Coater 108auto, Cressington). Using the FIB system, a trapezium-shaped precut (16 nA/30 kV) was performed and 50 nm thick tomography slices (1 nA/30 kV) covering the area of interest were cut subsequently (see Supporting Information File 1). The resulting stack of images shows the microstructure of the setal bases. One advantage of the FIB slicing technique is the possibility of identifying clubs and pins as such. This allows a target preparation of a specific setal base, which for light microscopy and TEM is almost impossible. The FIB analysis again showed evidence of mechanoreceptors at the setal bases and clear differences in the morphologies of clubs and pins (Supporting Information File 1).

### Lab-based µCT of the hemelytra

For a three-dimensional analysis of a whole, air-dried hemelytron of *N. glauca*, the hemelytron was scanned in a commercial µCT system (Skyscan 1272, Bruker Corp.) at 30 kV tube voltage, 212 µA tube current and with an effective pixel size of 3 µm.

### Synchrotron-µCT of basal hemelytra

More detailed cross sections of parts of a hemelytron were created at the Swiss Light Source synchrotron of the Paul Scherrer Institute (PSI; Villigen, Switzerland; X02DA TOMCAT beamline). The X-rays, produced by a 2.9 T superbending magnet on a 2.4 GeV storage ring (ring current = 400 mA, top-up mode), were monochromated with a double-multilayer monochromator and tuned to an X-ray energy of 12 keV. A scientific CMOS detector (pco.Edge 5.5) was used in combination with 10× magnifying visible-light optics (UPLAPO10x) and a 20 µm thick scintillator (LuAG:Ce), yielding an effective pixel size of 0.65 µm. A short sample-to-detector distance was used for obtaining an attenuation-contrast type mode and maximizing spatial resolution [19].

### Measurement of seta geometry and visualization of (SR-)µCT data

To study the three-dimensional architecture of the setae, the virtual (SR-)µCT sections were preprocessed in ImageJ 1.51u [20]. The gray-value based surface models of the hemelytron were created in InVesalius 3.0 [21]. Seta measurements and three-dimensional visualization were performed in Blender 2.79 (blender.org).

### Behavioral experiments

Twenty five individuals of *Notonecta* (mean body length 1.5 cm) were kept in small aquaria (4–5 individuals per tank). Tests were conducted in an experimental tank (19 × 7 cm^2^, water level 8 cm) and the animals were filmed (Sony DCR SR 55 with night shot function) under near infrared illumination (NIR, EcoLine IR Illuminator, wavelength 850 nm). A lux meter (PeakTech 5020) revealed 0 lux in the experimental room. During each trial, only one *Notonecta* was in the experimental tank. Three flat pieces of cork (4 × 5 cm^2^) were placed on the water surface and served as a resting plate for *Notonecta*. Control experiments revealed that *Notonecta* does not use its visual system under these conditions. Moreover, tests with small artificial fish never elicited a motor response, suggesting that only stimuli generated by real fish trigger the fish catching behavior.

Prior to an experiment, backswimmers were allowed to acclimatize for ≥30 minutes in the experimental tank. Thereafter a fish (body length 1.8–2.0 cm) was released into the tank. Video recordings started when the backswimmer rested under the cork piece. The cork piece excluded that fish could be detected by surface waves [22]. The evaluation of the videos was done with the program VidAna 2.01.

### Biomimetic proof of principle

In order to prove the functional principle of the *Notonecta* sensor, a technical air-retaining surface, comparable with the one previously described by Gandyra [23], was placed in a 30 × 20 × 25 cm^3^ experimental tank at a water depth of about 15 cm (Figure 8). The sample covered an area of 5 × 5 mm^2^ with an array of 3 × 3 needles of 3 mm height surrounded by a 3 mm high rim. A laser pointer (Logitech R800) was placed at one side of the experimental tank such that the beam reached the air–water interface at an angle of about 30°. The beam was reflected at the interface and impinged on a position-sensitive photodiode (PSD), placed at the opposite side of the experimental tank. Pressure changes caused a movement of the air–water interface and deflected the laser beam. The output voltage of the photodiode was used to monitor the deflection of the laser beam and thus the pressure changes. With this setup, we were able to record the conversation of two people standing in front of the experimental tank (Figure 8). To rule out the possibility that the recorded signals were caused by vibrations of the experimental tank, an experiment was performed replacing the air-retaining surface by a reflecting metal slide as well as a sample made of the same epoxy resin used for the air-retaining needle surface. In both cases no signal change was detected, which confirms the aforementioned considerations.

## Supporting Information

File 1 FIB tomography slices.
The video shows the tomography slices of a club-seta (left) and a pin-seta (right) recorded using the FIB technique. In the images, differences in the micromorphology of the two setae types can be seen.

File 2 Behavioral experiment.
Sample video of the behavioral experiments. A backswimmer, lurking in complete darkness under cork pieces, attacks a fish approaching from its back.
